# A deep learning-based image analysis model for automated scoring of horizontal ocular movement disorders

**DOI:** 10.3389/fneur.2025.1522894

**Published:** 2025-07-02

**Authors:** Xiao-lu Jin, Yu-fei Liu, Bing-bing He, Yi-fei Fan, Ling-yun Zhou

**Affiliations:** ^1^Ocular Motility Disorder Treatment and Rehabilitation Center, Department of Acupuncture, Harbin Medical University, Harbin, Heilongjiang Province, China; ^2^Ocular Motility Disorder Treatment and Rehabilitation Center, The First Affiliated Hospital of Harbin Medical University, Harbin, Heilongjiang Province, China

**Keywords:** ocular movement disorders, deep learning, artificial intelligence, automated scoring, ocular key points

## Abstract

**Introduction:**

This study proposes a deep learning–based image analysis method for automated scoring of the severity of horizontal ocular movement disorders and evaluates its performance against traditional manual scoring methods.

**Methods:**

A total of 2,565 ocular images were prospectively collected from 164 patients with ocular movement disorders and 121 healthy subjects. These images were labeled and used as the training set for the RetinaEye automatic scoring model. Additionally, 184 binocular gaze images (left and right turns) were collected from 92 patients with limited horizontal ocular movement, serving as the test set. Manual and automatic scoring were performed on the test set using ImageJ and RetinaEye, respectively. Furthermore, the consistency and correlation between the two scoring methods were assessed.

**Results:**

RetinaEye successfully identified the centers of both pupils, as well as the positions of the medial and lateral canthi. It also automatically calculated the horizontal ocular movement scores based on the pixel coordinates of these key points. The model demonstrated high accuracy in identifying key points, particularly the lateral canthi. In the test group, manual and automated scoring results showed a high level of consistency and positive correlation among all affected oculi (*κ* = 0.860, *p* < 0.001; *ρ* = 0.897, *p* < 0.001).

**Conclusion:**

The automatic scoring method based on RetinaEye demonstrated high consistency with manual scoring results. This new method objectively assesses the severity of horizontal ocular movement disorders and holds great potential for diagnosis and treatment selection.

## 1 Introduction

Ocular movement disorders refer to limitations in eye movement or fixed eye positions that result from damage to the ocular motor nerves or dysfunction of the neuromuscular junction, often accompanied by diplopia (1). These conditions considerably affect the patient’s quality of life. Therefore, accurate and objective assessment of the severity of ocular movement disorders is essential for diagnosing ocular motor nerve palsy, evaluating prognosis, and selecting appropriate treatment options. Clinically, the corneal reflex test, combined with a grading scale, is commonly used to assess the range of ocular movements (2). Our preliminary research indicated that manual measurement and comparison of the distances from the pupil center to the medial and lateral canthi in different gaze positions represents a reliable and clinically useful approach for scoring the severity of ocular movement disorders (3, 4). However, the accuracy of these two grading methods depends on the expertise of trained physicians, presenting subjectivity. The modified limbus test is a method that captures photographs of the nine cardinal gaze positions, sequentially overlays semi-transparent images of the secondary gaze positions onto the primary gaze position image, and measure the edge-to-edge distance. Then, geometric analysis is performed to calculate the angles of ocular rotation corresponding to each gaze position (5), though it requires manual measurement after overlaying two images, which can lead to issues such as imperfect alignment and measurement error.

Advancements in artificial intelligence for ocular movement analysis are rapidly progressing. Traditional machine learning models can generate classification models from ocular movement datasets (6). Deep learning models based on convolutional neural networks can automatically locate and segment the ocular region, facilitating the measurement of ocular movement distance in photographs of nine gaze positions (7). However, this model is limited to the localization and segmentation of the ocular region. It still requires manual input for distance measurement and has not been validated in patients with eyelid dysfunction or ocular motility disorders. Additionally, the results of automatic and manual measurements using through this method show significant differences, warranting caution in clinical practice. Therefore, we aim to explore a fully automated model for scoring the severity of ocular movement disorders to rapidly obtain accurate results.

RetinaFace is a high-precision face detection algorithm that employs self- and joint-supervised multi-task learning to perform pixel-level facial localization across faces of varying scales (8). Besides detecting the position of the facial bounding box, this algorithm can accurately localize key points of interest. Therefore, the present study aims to introduce an improved method based on RetinaFace to achieve orbital localization and detection of ocular key points. Furthermore, this approach will be integrated with our previously developed scoring scale for ocular movement disorders (3) to enable accurate and efficient automated scoring of ocular movement disorders.

## 2 Materials and methods

### 2.1 Study participants

A total of 164 patients with ocular motility disorders (including vertical and horizontal movement disorders) who visited the Ocular Motility Disorder Center at the First Affiliated Hospital of Harbin Medical University between January 2024 and October 2024 were prospectively enrolled. Additionally, 121 healthy volunteers were recruited. Photographs were obtained in nine gaze positions for all participants. Then, this training dataset was used to train the automatic scoring model.

A total of 92 patients with horizontal ocular motility limitations were also enrolled (these 92 patients were not included among the 164 patients in the training set). Photographs of each patient in leftward and rightward gaze positions were taken and used as the test set for the automatic scoring model. The exclusion criteria for the test group were patients with horizontal nystagmus or myogenic myasthenia gravis.

### 2.2 Ethics statement

This study was conducted in accordance with the principles outlined in *the Declaration of Helsinki* and was approved by the Ethics Committee of the First Affiliated Hospital of Harbin Medical University (No: 2024JS97), with informed consent obtained from each volunteer in advance.

### 2.3 Photography and image collection

Ocular images were collected by an experienced clinical ophthalmologist. Subjects’ chins and foreheads were stabilized on a headrest, with the head maintained at a horizontal position and secured with straps. A digital camera (G1X Mark II; Canon Inc., Japan) was positioned 50 cm in front of the eyes and aligned horizontally with the head. Subjects in the training set were directed to gaze at each of nine gaze positions (The directions are: up, down, left, right, upper left, upper right, lower left, lower right, and straight ahead.), with images captured at each position. In the test group, images were collected only when subjects gazed maximally to the left and right. In all subjects, when gazing downward or in cases of ptosis, the upper eyelid was gently lifted to improve the visualization of the pupil position.

### 2.4 Scoring criteria and calculation method

**Figure 1:**
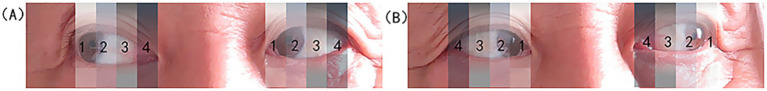
Horizontal ocular movement scoring diagram. **(A)** Scoring diagram for both eyes gaze to the right. **(B)** Scoring diagram when both eyes gaze to the left.

The scoring method for ocular movement disorders followed the scoring criteria in the ocular motor nerve palsy scale (3): 1 = affected eye movement is close to that of the healthy oculus, with the pupil center reaching or exceeding the ipsilateral 1/4 line in the paralyzed direction; 2 = the pupil center of the affected eye just crosses the midline but does not reach the ipsilateral 1/4 line in the paralyzed direction; 3 = the pupil center of the affected eye reaches or exceeds the contralateral 1/4 line in the paralyzed direction but does not reach or only reaches the midline; 4 = no movement toward the paralyzed side, or it does not reach the contralateral 1/4 line in the paralyzed direction. When both eye gazed to the right, the scoring included the abduction movement of the right eye and the adduction movement of the left eye. When both eyes gazed to the left, the scoring included the abduction movement of the left eye and the adduction movement of the right eye (Figure 1). Table 1 summarizes the calculation method for ocular movement disorder scores based on the aforementioned criteria.

**Table 1:** Scoring method for horizontal ocular movement disorders.

	Score	Ratio
Abduction movement	1	R1 ≤ 1/4
	2	1/4 < R1 ≤ 1/2
	3	1/2 < R1 ≤ 3/4;
	4	3/4 < R1
Adduction movement	1	R2 ≤ 1/4
	2	1/4 < R2 ≤ 1/2
	3	1/2 < R2 ≤ 3/4;
	4	3/4 < R2

R1 = (|Xpupil-Xtemporal|)/(|Xinner-Xtemporal|); R2 = (|Xpupil-Xinner|)/(|Xinner-Xtemporal|); Xpupil, Xtemporal, and Xinner represent the horizontal pixel coordinates of the pupil center point, the temporal canthus, and the inner canthus in the image, respectively.

### 2.5 Automatic scoring

#### 2.5.1 Eye feature point annotation

**Figure 2:**
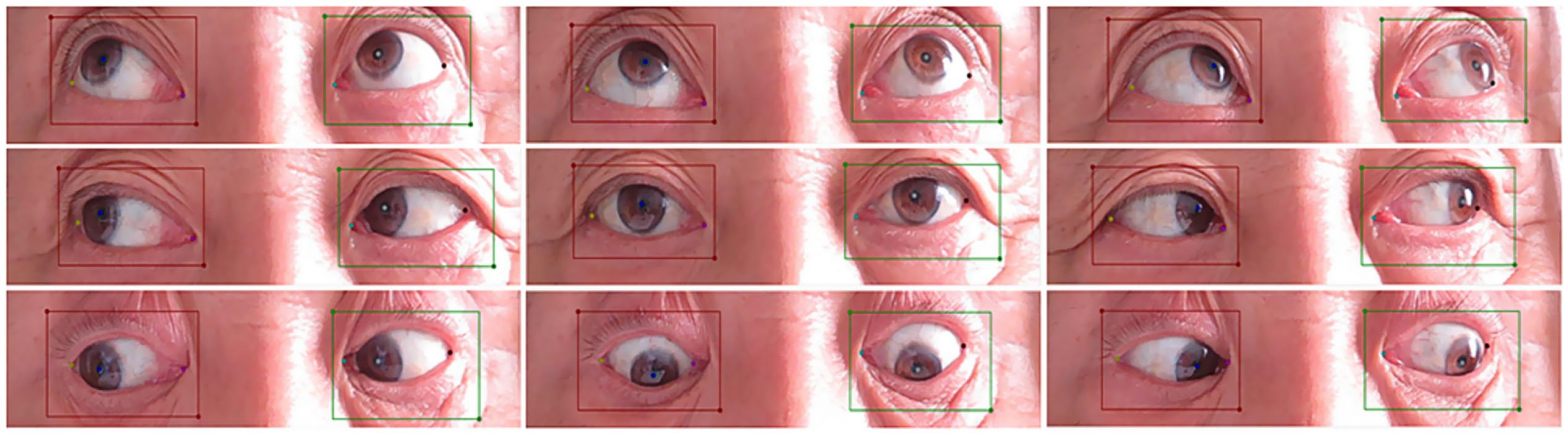
Annotated images of nine gaze positions.

An experienced ophthalmologists used Labelme software to annotate ocular images at nine gaze positions in the training group, marking the locations of both eye, pupil center points, medial canthi, and lateral canthi (Figure 2). The position of the pupil center if the cornea was occluded by the eyelid was determined through manual fitting. All annotation data were reviewed by another physician. In cases of ambiguous annotation points, decisions were made through discussion between the two physicians.

#### 2.5.2 Model development

**Figure 3:**
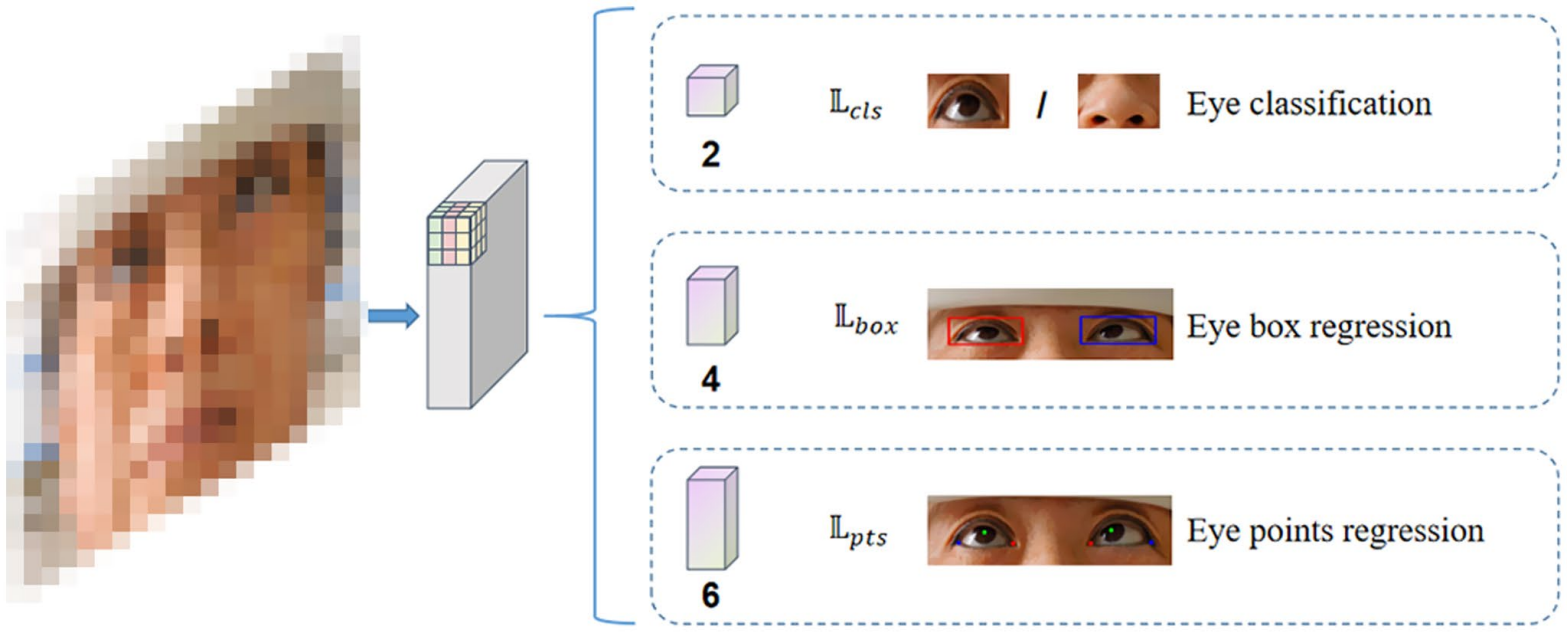
Overall architecture diagram of RetinaEye.

This study utilized a convolutional neural network–based RetinaFace detector architecture, adjusting the head network and improving data augmentation methods to suit the tasks of ocular detection and key point localization in this research. The overall architecture of RetinaEye included image feature extraction, classification, position detection, and key point localization multi-task learning (Figure 3).

Step 1 was image preprocessing. The images and annotation data of the training set were preprocessed using the OpenCV and Albumentations image processing libraries. The preprocessing operations included random cropping, aspect ratio padding, random rotation (within 40°), random brightness adjustment, aspect ratio scaling, and mean subtraction to augment the sample size.

**Figure 4:**
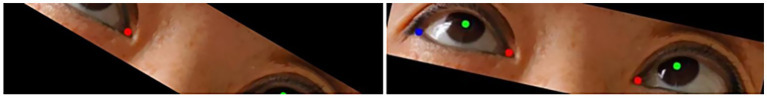
Key point loss due to rotation.

Random rotation may cause in the loss of key points (Figure 4). In such cases, the random rotation was performed again (up to three times) to ensure that the key points remained usable after rotation. Rarely, when an image still had no usable key points after 3 random rotations, it was considered unsuitable for random rotation operations; thus, the rotation step was skipped.

**Figure 5:**
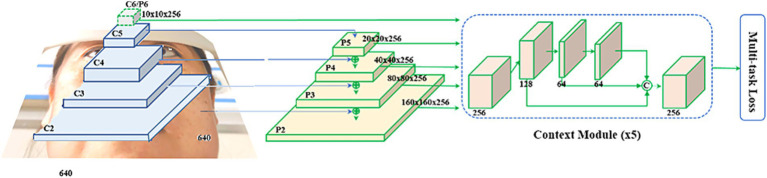
Feature extraction module.

Step 2 was establishing the RetinaEye algorithm structure. The algorithm structure of this study was based on the RetinaFace single-stage detector, with the feature extraction module built upon a feature pyramid that incorporated independent context modules, calculating multi-task loss for each anchor on the feature maps (Figure 5).

For each trained anchor i, its classification loss + bounding box regression loss + key point regression loss were calculated, as shown in Equation. 1.


(1) $$L_{\text{Total}}=L_{\mathrm{cls}}(p_{i},p_{i}^{*})+\lambda_{1}p_{i}^{*}L_{\mathrm{box}}(t_{i},t_{i}^{*})+\lambda_{2}p_{i}^{*}L_{\mathrm{pts}}(l_{i},l_{i}^{*})$$

where $p_{i}$ represents the probability predicted for the ocular region, while $p_{i}^{*}$ represents the ground truth. The formula shows, that for all negative sample anchors, only the classification loss is utilized. For positive sample anchors, the multi-task loss was calculated.

$L_{\mathrm{cls}}(p_{i},p_{i}^{*})$ denotes the classification loss for the ocular region, and $L_{\mathrm{cls}}$ uses the softmax binary classification loss function.

$L_{\mathrm{box}}(t_{i},t_{i}^{*})$ represents the bounding box regression loss for the ocular region, where $t_{i}=(t_{x},t_{y},t_{w},t_{h}$ ,) and $t_{i}^{*}=(t_{x}^{*},t_{y}^{*},t_{w}^{*},t_{h}^{*})$ denote the predicted bounding box position and the ground truth annotation position for the positive sample anchor, respectively. The regression function employs the ${\text{smooth}}_{L1}$ loss.

$L_{\mathrm{pts}}(l_{i},l_{i}^{*})$ represents the regression loss for ocular keypoints, where $l_{i}=(l_{x1},l_{y1},l_{x2},l_{y2},l_{x3},l_{y3}$ ,) and $l_{i}^{*}=(l_{x1}^{*},l_{y1}^{*},l_{x2}^{*},l_{y2}^{*},l_{x3}^{*},l_{y3}^{*})$ denote the predicted and ground truth values, respectively, for the outer canthus, pupil, and inner canthus points of the positive sample anchor.

The measurements of the three loss functions correspond to the outputs of the three different head networks illustrated in Figure 3: the classification of whether the current anchor region contains the ocular position, the output of the bounding box for foreground anchors, which include the ocular position; and the output of the key points for the ocular region.

Step 3 was training. The RetinaEye training set comprised 2,565 images with corresponding annotation information. Training was conducted on a server equipped with an Intel Xeon 8-core processor, a Tesla P40 GPU, and 64 GB of memory, running the Ubuntu 20.04 operating system. The training utilized the Python programming language and the PyTorch deep learning framework, with the SGD optimizer selected. The initial learning rate was set to $10^{-3}$ , which was increased to $10^{-2}$ after 5 epochs. Subsequently, the learning rate was reduced to one-tenth of its original value at epochs 40 and 50, with training concluded at epoch 80.

Step 4 was automatic scoring. The images of patients gazing left and right in the test group were automatically scored according to the established scoring criteria.

### 2.6 Manual scoring

A clinically experienced ophthalmologist manually marked the positions of the pupil center, medial canthus, and lateral canthus in the test group images for left and right gazes using ImageJ software. The scoring was performed based on the horizontal coordinates of these three points according to the ocular movement scoring calculation method.

### 2.7 Statistical analysis

The mean Euclidean distance (MED) was calculated as the average distance between the pixel coordinates of the key points identified by the model and the pixel coordinates of the manually annotated points to assess the accuracy. A smaller average distance indicated higher accuracy. The weighted kappa coefficient (WK) was calculated to evaluate the consistency between manual and automatic scoring. If WK = 1, it indicated complete agreement between the two methods; 0.75 ≤ WK < 1 was generally considered high consistency; 0.4 ≤ WK < 0.75 indicated moderate consistency; 0 < WK < 0.40 represented low consistency; and WK = 0 signified complete disagreement. The Spearman correlation coefficient was calculated to assess the correlation between the results obtained from the two scoring methods. All statistical analyses were conducted using SPSS version 25.0, with *p* < 0.05 indicating statistical significance.

## 3 Results

The training group included a total of 285 participants, with 2,565 ocular images collected. The testing group initially included 94 patients with horizontal ocular movement disorders; however, 2 patients were excluded for refusing to sign the informed consent, resulting in a final total of 92 participants with 184 ocular images. Among them, 25 patients had adduction movement disorders and 67 had abduction movement disorders, comprising 26 eyes with adduction disorders and 73 eyes with abduction disorders. All patients were of Asian ethnicity. The basic demographic information of participants in the training and testing groups is shown in Table 2.

**Table 2:** Basic demographic information of participants in the training and testing groups.

		Training group	Testing group
Total number of participants (*n*)		285	92
Mean age (years ± SD)		55.89 ± 9.30	60.9 ± 12.2
Male (%)		61.8%	65.2%
Different types (*n*)	Horizontal movement disorder	97	92
	Vertical movement disorder	32	0
	Horizontal and vertical movement disorder	35	0
	Healthy	121	0
Different etiologies (*n*)	Oculomotor nerve palsy	62	25
	Trochlear nerve palsy	11	0
	Abducens nerve palsy	79	67
	Mixed nerve palsy	4	0
	Thyroid ophthalmopathy	5	0
	Others	3	0

### 3.1 Key point recognition results

**Figure 6:**
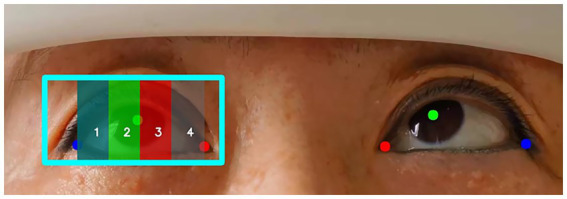
Example of automatic scoring output for ocular abduction. Blue box: position of the right eye; red dot: medial canthus; green dot: pupil center; blue dot: lateral canthus. The position of the pupil center in different regions (1, 2, 3, 4) represents the movement scores for the abduction of the right eye.

After training using the nine gaze positions from the training group, RetinaEye was tested on images from the test group. The model successfully detected the positions of both eyes, identifying their pupil centers, medial canthi, and lateral canthi. It also output the coordinates of the three key points and automatically calculated the scores for abduction and adduction movement disorders of both eyes. Figure 6 illustrates a representative example of the automatic scoring output for the abduction of the right eye.

### 3.2 Results of key point recognition accuracy by the model

Table 3 shows the MED between the pixel coordinates of key points (The pupil center point, inner canthus point, and outer canthus point identified by the model) identified by the model and those of manually annotated points. The average Euclidean distance for the lateral canthi of both eyes was relatively small, while that for the medial canthi was somewhat larger, indicating that the model performed well in predicting the lateral canthi but was slightly less accurate in predicting the medial canthi. The average Euclidean distances for all three key points of the right eye were smaller than those of the left eye, suggesting that the model achieved greater accuracy in predicting the coordinates of the left eye key points compared to the right eye.

**Table 3:** Mean Euclidean distance of different key points.

Unit: pixel			
Eye	Medial canthus ( $\bar{d},\bar{X\;}$ ± s)	Pupil center ( $\bar{d},\bar{X\;}$ ± s)	Lateral canthus ( $\bar{d},\bar{X\;}$ ± s)
Left eye	11.777 ± 0.879	9.661 ± 0.719	5.047 ± 0.470
Right eye	10.366 ± 0.983	8.590 ± 1.086	4.957 ± 0.482

$\bar{d\;}$ represents the mean Euclidean distance; $\bar{X\;}$ ± s represents the mean ± standard deviation.

### 3.3 Consistency between manual and automatic scoring results

After scoring the adduction and abduction movements of both eyes in the test set images using manual annotation and automatic prediction methods, the weighted kappa coefficients between the manual and automatic scoring results were calculated (Table 4). The consistency results indicate a high degree of agreement between the two measurement methods (*κ* = 0.857, *p* < 0.001), particularly in eyes with adduction movement disorders, where the consistency was highest. The Spearman correlation coefficient between the two scoring methods was as *ρ* = 0.897 (*p* < 0.001), demonstrating a strong positive correlation between the results obtained from both methods.

**Table 4:** Consistency results between manual scoring and automatic scoring.

	Weighted kappa coefficient (*κ*)	Pearman correlation coefficient (*ρ*)
All affected eyes	0.857^*^	0.897^*^
Eyes with abduction movement disorders	0.835^*^	0.864^*^
Eyes with adduction movement disorders	0.912^*^	0.899^*^

*p < 0.001.

## 4 Discussion

This study proposes a deep learning model named RetinaEye. Trained on ocular photographs with manually annotated key points, this model accurately identified the pixel coordinates of the orbit, pupil centers, medial canthi, and lateral canthi of both eyes. Additionally, it automatically calculated scores for ocular movement disorders.

Clinically, multiple traditional methods exist to assess ocular movements; however, grading the severity of movement disorders remains challenging. The corneal light reflex test, a common clinical method, instructs the subject to maintain a steady head position while attempting the maximal gaze in nine directions. Then, the positions of the eyes and corneal light reflex are observed, allowing for the assessment of ocular movements, with scores ranging from −1 to −4 or +1 to +4 (2). Additionally, corneal limbus measurement (9) is a convenient and efficient assessment method. It involves placing a transparent ruler directly in front of the cornea to measure the movement distance of the limbus when the ocular shifts from the first gaze position to the second or third positions, allowing for the evaluation of ocular motility. Despite being straightforward, non-invasive, and easy to perform, the accuracy of these methods largely depends on the clinician’s experience. Additionally, the corneal light reflex test is less applicable in patients with corneal damage, and the limbus measurement has limited precision. Other methods that quantify ocular movement angles, such as manual perimetry, scleral search coils, and synoptophore, have drawbacks, including extended measurement time, high costs, and limited measurement ranges (7).

Photographic eye assessment is increasingly used to evaluate ocular movements. Photos taken in various gaze positions can be used to assess the severity of abduction dysfunction (10) and oblique muscle dysfunction (11, 12) for scoring. Compared to traditional assessments, photography offers greater objectivity, although the photo analysis can be labor-intensive and slow when processing large volumes of images. Artificial intelligence applications have enabled the rapid analysis of such images for ocular movement assessment. For instance, an application using ResNet-50 as its neural network architecture was trained on facial photographs to classify images into nine gaze positions (13). Zheng (14) developed a deep learning model that identifies horizontal strabismus from primary gaze images, achieving diagnostic performance comparable to, or exceeding, that of ophthalmologists. These models lay the foundation for early detection of horizontal strabismus and for distinction between leftward and rightward gaze images, facilitating subsequent ocular movement scoring. Lou (7) developed an image analysis method using deep learning to automatically measure ocular movement distance based on nine gaze photos, aiding in the evaluation of the range of ocular movements. However, this model does not provide automated grading for the range of movements.

Therefore, this study introduces RetinaEye, which can achieve rapid, objective, and automated scoring of ocular movement disorders. RetinaEye demonstrated overall excellent performance in key point recognition, although its accuracy varied across different key points. A comparison of left and right eye key point predictions showed that RetinaEye achieved slightly higher accuracy for the right oculus in predicting the coordinates of the pupil center ( $\bar{d}$ = 8.590 ± 1.086), medial canthus ( $\bar{d}$ = 10.366 ± 0.983), and lateral canthus ( $\bar{d}$ = 4.957 ± 0.482), compared to the left ocular pupil center ( $\bar{d}$ = 9.661 ± 0.719), medial canthus ( $\bar{d}$ = 11.777 ± 0.879), and lateral canthus ( $\bar{d}$ = 5.047 ± 0.470). Additionally, the prediction accuracy of RetinaEye for the medial canthi of both eyes was lower than for the pupil center and lateral canthus. This might stem from the common presence of epicanthus in Asians, which shortens the horizontal distance between the eyes (15) and may lead to annotation and prediction discrepancies in the medial canthus.

The manual and automated scoring results also showed high consistency, particularly in scoring adduction disorders, with a weighted kappa coefficient of 0.912 (*p* < 0.001). The Spearman correlation coefficient was 0.897 (*p* < 0.001), further demonstrating a strong correlation between the two and indicating that RetinaEye can provide clinicians with a reliable automated tool to reduce workload and improve diagnostic efficiency, especially in cases requiring large-scale photo analysis. This outcome provides robust support for artificial intelligence–based automated assessment of ocular movement disorders.

However, this study has some limitations. First, it only addresses horizontal movement scoring without evaluating vertical movement disorders. Vertical movement assessment involves additional anatomical complexities, including the positions of the lower edge of the pupil and the lower eyelid, posing greater demands on the model. Future research should expand on this foundation to achieve comprehensive automated scoring for ocular movement disorders. Secondly, the model’s mean Euclidean distance in predicting medial canthus positions was relatively high, suggesting room for further improvement in key point recognition. Additionally, when calculating horizontal ocular movement scores, this study did not fully account for the vertical disparity between the medial and lateral canthi, which, though minor, may slightly affect scoring accuracy.

Future research should expand the scope of this model, exploring its performance across different races, age groups, and in vertical movement disorders. The model’s potential for remote application is also highly promising. With further refinements, it may facilitate remote ocular selfies for diagnostic purposes, reducing healthcare costs for patients and providing valuable resources, particularly in remote or underserved areas.

## 5 Conclusion

This study presents a deep learning model named RetinaEye, which can automatically analyze ocular photographs taken during horizontal gaze positions. The model accurately identifies the positions of the orbit, pupil center, inner canthus, and outer canthus, and calculates scores for ocular movement disorders. A comparison with traditional manual scoring results revealed a high correlation and strong consistency between the two methods. These findings strongly support the use of artificial intelligence for the automated assessment of ocular movement disorders.

## Data Availability

The raw data supporting the conclusions of this article will be made available by the authors, without undue reservation.
